# Epidemiology of stroke in Shiraz, Iran

**Published:** 2015-07-06

**Authors:** Babak Daneshfard, Sadegh Izadi, Abdolhamid Shariat, Mohammad Amin Toudaji, Zahra Beyzavi, Leila Niknam

**Affiliations:** 1 Research Center for Traditional Medicine and History of Medicine AND Essence of Parsiyan Wisdom Institute, Traditional Medicine and Medicinal Plant Incubator, Shiraz University of Medical Sciences, Shiraz, Iran; 2 Shiraz Neuroscience Research Center AND Department of Neurology, Shiraz University of Medical Sciences, Shiraz, Iran; 3 Shiraz Neuroscience Research Center AND Clinical Neurology Research Center AND Department of Neurology, Shiraz University of Medical Sciences, Shiraz, Iran; 4 Shiraz Neuroscience Research Center AND Student Research Committee, Shiraz University of Medical Sciences, Shiraz, Iran

**Keywords:** Stroke, Cerebrovascular Disorders, Epidemiology, Shiraz

## Abstract

**Background:** Stroke is the main cause of physical disability and the second leading cause of death worldwide. Two-thirds of all strokes occur in the developing countries. Despite being preventable, stroke is increasingly becoming a major health issue in these countries. The aim of this study was to evaluate the epidemiology of stroke in Shiraz, Iran, one of the main referral centers in the southwestern part of Iran.

**Methods: **A cross-sectional study was conducted on all stroke patients admitted to the Namazee Hospital, affiliated to Shiraz University of Medical Sciences, between August 2010 and January 2011. Patients’ demographic data, atherosclerosis risk factors, type of stroke, drug history, outcomes, and neurological signs were recorded. Chi-square test, Kolmogorov–Smirnov test, t-test, and Mann–Whitney U-test were used to analyze the data.

**Results: **A total of 305 patients with stroke, aged 27-97 years (mean ± SD = 68.33 ± 12.99), 269 patients (88.2%) had ischemic stroke (IS) and 36 (11.8%) had hemorrhagic stroke (HS). 133 patients (43.6%) were men and 172 (56.4%) were women. 11.4% of the patients with IS and 40.6% with HS died during hospitalization, causing 12.1% death in all stroke patients [Odds ratio (Or) = 5.34, 95% Confidence intervals (CI) = 2.35-12.11]. Hypertension, ischemic heart disease, diabetes, and recurrent stroke were the most common risk factors.

**Conclusion:** This study provides evidence that the epidemiology of stroke in the southwestern part of Iran may be similar to other places. However, it seems necessary and helpful to design a registration system for patients with stroke in Shiraz Namazee Hospital.

## Introduction

According to the World Health Organization, stroke is the rapid progression of signs and symptoms, caused by limited or widespread disruption of brain function, that has vascular origin and takes more than 24 h.^1^^,^^2^ Stroke can be generally divided into two categories: Ischemic stroke (IS) and hemorrhagic stroke (HS).^1^

Stroke is the second leading cause of death worldwide which is considered as the third one in the United States and other industrialized countries.^3^^-^^8^ Each year, 55 million deaths occur in the world that 10% of them are due to the stroke.^9^ In the United States, about 780,000 strokes occur each year (one in every 40 s) while 87% are IS and 13% are HS.^9^^-^^11^ Annual mortality of the disease in this country is 150,000 people (one in every 4-3 min), so it is estimated that one out of every 16 Americans dies due to stroke.^9^

The deaths occurring within 28 days after the stroke in the Middle East and North Africa vary from 10% in Kuwait to 31.5% in Iran.^4^ Two-thirds of all strokes occur in the developing countries which, in spite of their preventable nature, are increasingly becoming a major health problem.^12^^,^^13^ It is expected that the deaths resulting from stroke will nearly double in the Middle East and North Africa by 2030.^4^ A major risk factor for the stroke is increasing age as every 10 years after age 55 the risk of stroke doubles.^8^ Another risk factor is high blood pressure, which is the most common preventable cause of the disease.^11^ Other risk factors are diabetes, smoking, obesity, lack of exercise, taking a diet which is high in cholesterol and salt, alcohol, atrial fibrillation, family history, and oral contraceptive pill usage.^7^^,^^11^^,^^14^^,^^15^ In addition, gender is a determinant factor in this disease. In general, stroke is more common in men. However, because of the longer life expectancy for women and a high incidence of stroke in the older ages, the number of women with stroke is higher than men.^16^

Stroke, as the main cause of physical disability worldwide, is one of the main reasons for prolonged hospital stay that can lead to a significant increase in the cost of treatment.^7^^,^^8^ The direct and indirect cost of the stroke in the United States was 65.5 billion in 2008.^17^

A few studies conducted in Iran reported that the incidence of stroke is about 43 patients per 100,000 population.^18^ In a population-based study conducted in Mashhad, Iran, IS was 81.9% and HS was 15.1% of all the patients.^1^ The most common risk factor was high blood pressure with a prevalence rate of 54%.^18^^,^^19^ Incidence of stroke was slightly higher in women in all age groups (51-53%). However, in the age group of 15-45 years, stroke was more common in men, while the average age of its incidence is in the seventh decade of life. The hospital-based 28 days case fatality rate is reported at 19.2%^20^ and 31.5%^21^ in Iran. Another study refers to an unknown situation of this disease in the Middle East, that mismatch with data in the Western Countries that once again shows the need for more studies in this regard.^1^

One of the few studies conducted in Shiraz, Iran, in this field investigated early brain hemorrhage due to high blood pressure in patients referring to the hospitals of Shiraz University of Medical Sciences during 2002-2004.^22^ Another retrospective study investigated the documents of 16351 patients with stroke from 2001 to 2010 in Shiraz.^23^ Regarding the preventable nature of the disease, it is necessary to do more studies to determine the risk factors and the underlying causes in a particular population in order to outline and plan to prevent it.^18^

Considering that few epidemiological studies have been previously conducted in Shiraz, we conducted this study in Shiraz Namazee Hospital as a referral center for patients with stroke in the Fars province and southwestern part of Iran to obtain general information about the status of the disease in this region.

## Materials and Methods

This prospective cross-sectional study was conducted in Shiraz Namazee Hospital between August 2010 and January 2011. All patients with stroke, who were diagnosed based on their clinical manifestations and imaging (magnetic resonance imaging or computerized tomography scan), were included in the study and the patients with transient ischemic attack were excluded. Patients’ demographic data, atherosclerosis risk factors, type of stroke, drug history, neurological signs, duration of admission, and final outcomes were recorded.

SPSS software for Windows (version 16, SPSS Inc., Chicago, IL, USA) was used for the statistical analysis of the data. Chi-square test was used for the comparison between categorical variables and Kolmogorov–Smirnov test was used to report normally distributed quantitative data. In the case of normal variables, t-test and Mann–Whitney U-test were employed. P < 0.050 was considered statistically significant.

## Results

A total of 305 patients were included, aged between 27 and 97 years (mean ± SD = 68.33 ± 12.99). 7.9% of patients had ages of 45 or less. 133 patients (43.6%) were men and 172 (56.4%) were women. The age of most of them was between 61 and 80 years. 269 patients (88.2%) had IS and 36 (11.8%) had HS. The mean age of the patients with IS was 66.84 ± 16.94 and those with HS was 66.22 ± 12.14. 64 patients (21%) had a recurrent stroke. Data analysis did not reveal a statistically significant difference between mortality rates in the age groups (P = 0.993) (Table 1).

**Table 1:** Age groups and mortality rates in the patients, admitted to Shiraz Namazee Hospital, 2010-2011

**Age group ** **(year)**	**Frequency ** **(%)**	**Mortality (%) within ** **age group**
≤ 40	8 (2.6)	2 (25.0)
41-50	21 (6.9)	2 (9.5)
51-60	60 (19.7)	7 (11.7)
61-70	72 (23.6)	9 (12.5)
71-80	81 (26.6)	9 (11.1)
≥ 81	56 (18.4)	8 (14.3)
Missing	7 (2.3)	-
Total	305 (100)	37 (12.1)

About 12.1% of all the patients died during the hospitalization. 11.4% of the patients with IS and 40.6% with HS died [Odds ratio (OR) = 5.34, 95% Confidence intervals (CI) = 2.35-12.11]. Although the difference in the mortality rate was not statistically significant (P = 0.362), the rate was higher in men (17.4%) than in women (13.3%). Sex and age-adjusted OR for the mortality rate between the patients with HS in comparison and those who had IS was 5.30 (95% CI = 2.32-12.09).

**Figure 1:**
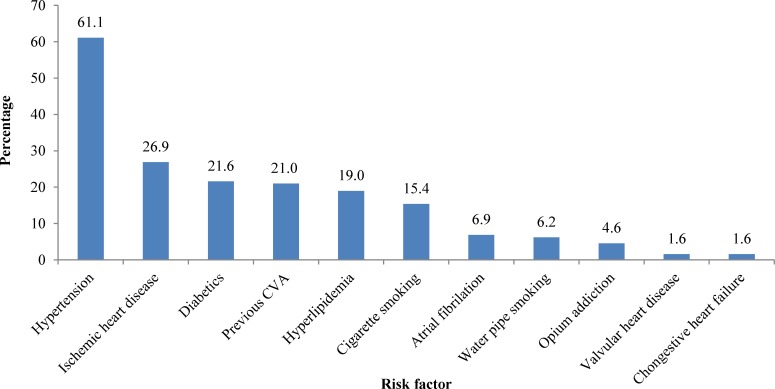
Prevalence of risk factors in the patients with stroke, admitted to Shiraz Namazee Hospital, 2010-2011

**Figure 2:**
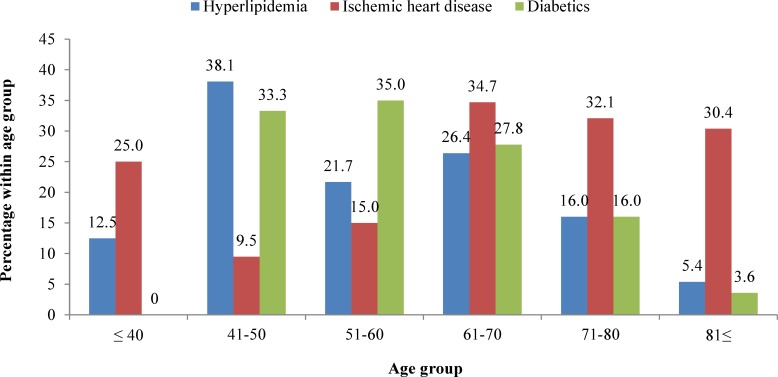
Risk factors in age groups of the patients with stroke, admitted to Shiraz Namazee Hospital, 2010-2011

Hypertension, ischemic heart disease, diabetes, and recurrent stroke were the most common risk factors (Figure 1). The prevalence of hyperlipidemia, ischemic heart disease, and diabetes was significantly different between the age groups. Hyperlipidemia, diabetes, and ischemic heart disease were more common in age groups of 41-50, 41-60 and above 60, respectively (Figure 2). There was no significant relationship between the risk factors and mortality of the patients.

**Figure 3:**
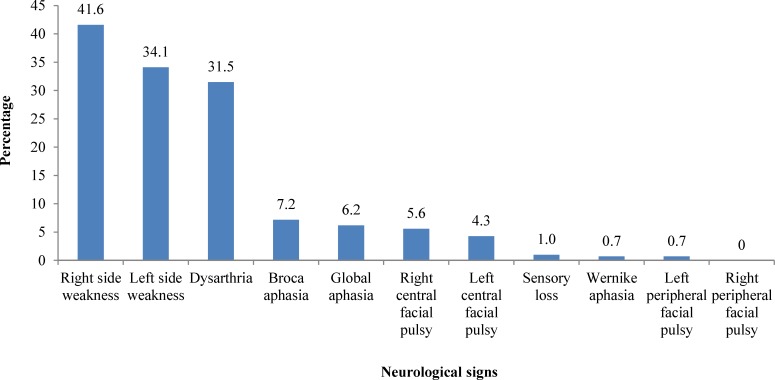
Prevalence of different neurological signs in the patients with stroke, admitted to Shiraz Namazee Hospital,  2010-2011

The most common neurological signs were hemiparesis and dysarthria (Figure 3). In general, there was no significant relationship between neurological signs and the mortality rate except for dysarthria. The patients with dysarthria had significantly less mortality (P = 0.019).

Three risk factors had different prevalence rates in the age groups: hyperlipidemia (P = 0.010), ischemic heart disease (P = 0.480), and diabetes (P < 0.001).

**Figure 4:**
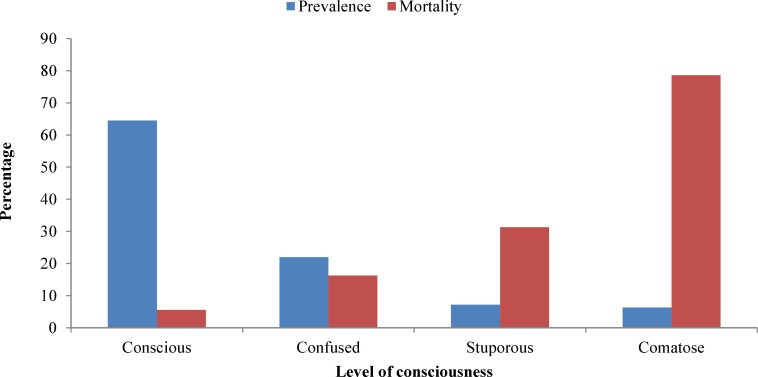
Relation between the level of consciousness and mortality in the patients with stroke, admitted to  Shiraz Namazee Hospital, 2010-2011

Data analysis showed the lower the level of consciousness is, the higher the mortality will be (P < 0.001)

There was a reverse relation between the level of consciousness and mortality rate (P < 0.001) (Figure 4). Mean of systolic blood pressure was higher in the patients with HS than IS (160 mmHg vs. 145 mmHg, P = 0.006). The mean of diastolic blood pressure of the patients with HS was higher than those with IS as well (90 mmHg vs. 83 mmHg, P = 0.013). Median length of hospital stay was 2 days for both types of strokes, the discharged and expired patients.

## Discussion

This study describes the epidemiology of stroke in Shiraz Namazee Hospital as an important referral center for the patients with stroke in the southwestern part of Iran. Our findings are in line with the findings reported by other studies. Proportion of the patients with IS and those who had HS in this study was 88.2% and 11.8%, respectively, which is comparable with the results of a population-based study conducted in Mashhad.^1^ The finding reported by Azarpazhooh et al.^1^ is also similar to the prevalence of the types of stroke in the United States.^6^^,^^11^^,^^17^ However, the prevalence of IS was less in Argentina and Latin America.^9^

Similar to the findings reported by other studies in Iran and the USA, the mean age of patients with stroke in our study was 68.3.^16^^,^^18^ Regarding the sex pattern of stroke in previous studies conducted in Iran,^18^ the present study confirms that women are more likely to experience stroke than men, but some studies have documented that 55% of the patients with stroke are male in the USA.^7^ It is difficult to explain this difference, but it might be related to different types of studies. However, similar to other studies,^2^ we found no sex difference in stroke mortality.

In our study, the mortality of different types of stroke in the average 2 days of hospitalization after stroke incidence is similar to 28 days mortality of other studies conducted in Iran and the USA.^11^^,^^17^^,^^18^ Nevertheless, the whole mortality in our study (12.1%) is less than what has been reported by other studies.^2^^,^^18^ This might be because of different study designs and the fact that, despite the others, we just considered the hospital course of the patients in their follow-up. However, it was higher than 28 days stroke mortality in our neighbor country, Kuwait.^4^ In addition, although HS is less prevalent than IS, its fatality is considerably higher.^11^ In the present study, we showed that HS was five times more fatal than IS.

Similar to our study, investigations in Iran and other countries show that the hypertension is the most prevalent risk factor for stroke.^9^^,^^11^^,^^18^ Ischemic heart disease and diabetes are the second risk factors, but other studies show that smoking is the third prevalent risk factor in Iran and the second one in Argentina and Latin America.^9^^,^^18^ A possible explanation for this difference might be due to the fact that we separated cigarette smoking, water pipe smoking, and opium addiction from each other.

Our findings showed that right and left side weakness and dysarthria are the most common neurological signs, which are in agreement with previous findings.^6^ An important finding of the present study was that both systolic and diastolic blood pressures were significantly higher in the patients who had HS that shows that the control of hypertension plays an important role in the reduction of stroke mortality.

There are several limitations in this study. First, it was a hospital-based study that has less accuracy in comparison with population-based studies. Second, the source of our data was patients’ documents that because of their defects, some data missing happened. Third, some case missing occurred due to the difficulties in coordination between different admission wards. We suggest that more detailed future population-based studies may be warranted for better healthcare planning in this regard and to further investigate the other aspects like economical and psychosocial burden of stroke.

## Conclusion

This study showed that the epidemiology of stroke in the southwestern part of Iran is similar to other places. However, because stroke is a serious health problem, there is an urgent need to design a stroke registration system in Shiraz for a better health planning. In addition, in the realm of prevention, our emphasis is on better control of hypertension to decrease the burden of stroke

## Notes:

**How to cite this article: **Daneshfard B, Izadi S, Shariat A, Toudaji MA, Beyzavi Z, Niknam L. Epidemiology of stroke in Shiraz, Iran. Iran J Neurol 2015; 14(3): 158-63.
